# Trends in Stimulant Prescription Fills Among Commercially Insured Children and Adults — United States, 2016–2021

**DOI:** 10.15585/mmwr.mm7213a1

**Published:** 2023-03-31

**Authors:** Melissa L. Danielson, Michele K. Bohm, Kimberly Newsome, Angelika H. Claussen, Jennifer W. Kaminski, Scott D. Grosse, Lila Siwakoti, Aziza Arifkhanova, Rebecca H. Bitsko, Lara R. Robinson

**Affiliations:** ^1^Division of Human Development and Disability, National Center on Birth Defects and Developmental Disabilities, CDC; ^2^Office of Policy Analytics & Population Health, Office of the Associate Director for Policy and Strategy, CDC; ^3^Office of the Director, National Center on Birth Defects and Developmental Disabilities, CDC.

Prescription stimulant use, primarily for the treatment of attention-deficit/hyperactivity disorder (ADHD), has increased among adults in the United States during recent decades, while remaining stable or declining among children and adolescents (*1*,*2*). MarketScan commercial claims data were analyzed to describe trends in prescription stimulant fills before and during the COVID-19 pandemic (2016–2021) by calculating annual percentages of enrollees aged 5–64 years in employer-sponsored health plans who had one or more prescription stimulant fills overall and by sex and age group. Overall, the percentage of enrollees with one or more prescription stimulant fills increased from 3.6% in 2016 to 4.1% in 2021. The percentages of females aged 15–44 years and males aged 25–44 years with prescription stimulant fills increased by more than 10% during 2020–2021. Future evaluation could determine if policy and health system reimbursement changes enacted during the pandemic contributed to the increase in stimulant prescriptions. Stimulants can offer substantial benefits for persons with ADHD, but also pose potential harms, including adverse effects, medication interactions, diversion and misuse, and overdoses. Well-established clinical guidelines exist for ADHD care, but only for children and adolescents* (*3*); clinical practice guidelines for adult ADHD could help adults also receive accurate diagnoses and appropriate treatment.

CDC analyzed claims data from the Merative MarketScan Commercial Database, a national convenience sample of deidentified health care claims from enrollees in employer-sponsored insurance plans. CDC accessed 2016–2021 MarketScan data using Treatment Pathways 4.0, an online analytic platform that includes plans with complete data on prescription drug fills, to calculate the annual percentages of persons continuously enrolled throughout the calendar year with one or more prescription stimulant^†^ fills. All prescription stimulants were included in the analyses, regardless of whether the enrollee had any claims with an ADHD diagnosis code present. Percentages and annual percent change (APC) were calculated for enrollees aged 5–64 years overall and by sex and age group; primary results were calculated by 5-year age groups, but some results were summarized by wider age groups to describe broader patterns. Among persons with one or more prescription stimulant fills during the calendar year, the mean number of prescription stimulant fills during that year and the percentage of persons who met a case definition for receipt of care for ADHD^§^ were calculated. Statistical testing was not performed because the size of the MarketScan database often results in significant p-values that are not clinically meaningful. All point estimates are presented, and changes >10% are highlighted. This activity was reviewed by CDC and was conducted consistent with applicable federal law and CDC policy.^¶^

Across all years, the percentages of male and female enrollees with one or more prescription stimulant fills were highest among those aged 5–19 and 15–24 years, respectively. Overall, the percentage of enrollees with prescription stimulant fills increased from 3.6% in 2016 to 4.1% in 2021, with percentages and APC varying by sex and age (Table) (Figure 1) (Figure 2). During 2016–2020, percentages remained stable or decreased among females aged ≤24 years (average APC range = −1.8% to 0.1%) and increased modestly among those aged 25–64 years (average APC range = 2.3% to 6.6%). However, during 2020–2021, the percentage of females with one or more prescription stimulant fills increased substantially among most age groups, with the largest changes among those aged 15–44 and 50–54 years (APC range = 14.3% to 19.2%).

**TABLE:** Percentage of persons aged 5–64 years with at least one stimulant prescription fill, by sex, age group, calendar year, average annual percent change (2016–2020), and annual percent change (2020–2021) — MarketScan commercial databases, United States, 2016–2021

Sex and age group, yrs	Percentage, by year						Average annual % change,* 2016–2020	Annual % change,* 2020–2021
	2016	2017	2018	2019	2020	2021		
**Sample size (millions)**	**20.7**	**19.0**	**17.4**	**16.0**	**15.6**	**13.3**	**—**	**—**
**Both sexes, all ages**	**3.6**	**3.7**	**3.6**	**3.7**	**3.8**	**4.1**	**1.4**	**7.9**
**Female, all**	**3.2**	**3.3**	**3.3**	**3.4**	**3.6**	**4.1**	**3.0**	**13.9**
5–9	3.0	3.0	2.9	2.9	2.9	2.9	−0.8	0
10–14	4.8	4.9	4.7	4.8	4.8	5.2	0	8.3
15–19	5.3	5.2	4.9	5.1	5.3	6.1	0.1	15.1
20–24	5.6	5.5	5.2	5.1	5.2	6.2	−1.8	19.2
25–29	4.2	4.4	4.4	4.5	4.6	5.4	2.3	17.4
30–34	3.5	3.8	3.9	4.1	4.4	5.1	5.9	15.9
35–39	3.1	3.4	3.5	3.7	4.0	4.7	6.6	17.5
40–44	3.0	3.1	3.1	3.3	3.5	4.0	4.0	14.3
45–49	2.6	2.8	2.9	3.0	3.2	3.5	5.3	9.4
50–54	2.1	2.2	2.2	2.4	2.5	2.9	4.5	16.0
55–59	1.6	1.7	1.7	1.8	1.9	2.0	4.4	5.3
60–64	1.2	1.3	1.3	1.4	1.4	1.5	4.0	7.1
**Male, all**	**3.9**	**4.0**	**4.0**	**4.0**	**4.0**	**4.2**	**0.6**	**5.0**
5–9	7.3	7.3	7.0	7.1	6.8	6.7	−1.7	−1.5
10–14	10.8	10.9	10.6	10.7	10.2	9.9	−1.4	−2.9
15–19	7.9	7.8	7.5	7.4	7.2	7.1	−2.3	−1.4
20–24	5.6	5.5	5.2	5.0	4.8	5.0	−3.8	4.2
25–29	4.0	4.1	4.1	4.1	4.2	4.7	1.2	11.9
30–34	3.4	3.6	3.8	3.9	4.1	4.7	4.8	14.6
35–39	2.7	2.9	3.0	3.3	3.4	3.9	6.0	14.7
40–44	2.1	2.2	2.3	2.5	2.7	3.0	6.5	11.1
45–49	1.7	1.8	1.9	2.1	2.1	2.3	5.5	9.5
50–54	1.3	1.4	1.4	1.5	1.6	1.8	5.4	12.5
55–59	1.0	1.0	1.1	1.1	1.2	1.3	4.8	8.3
60–64	0.9	0.9	0.9	0.9	0.9	0.9	0	0

* Annual percent change is calculated as the difference between percentage in one year and that in the preceding year, divided by the previous year’s percentage.

**FIGURE 1:**
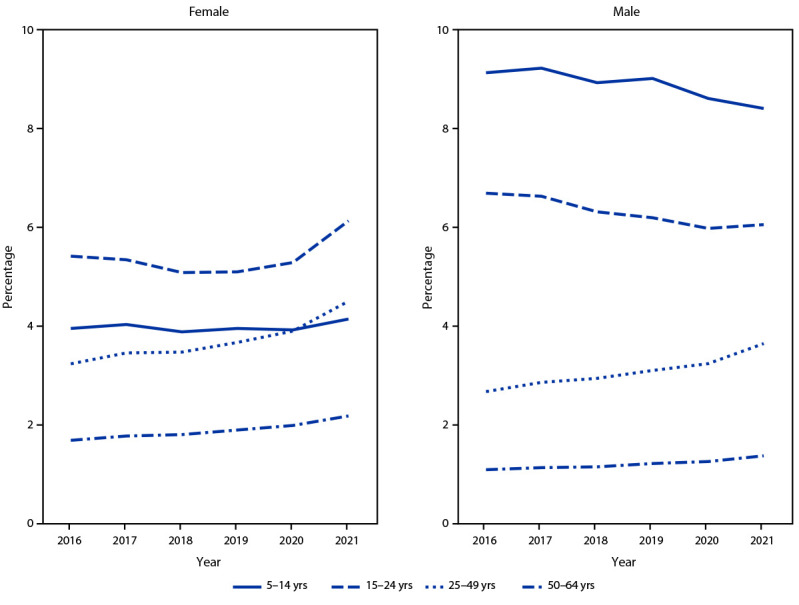
Percentage of persons aged 5–64 years with at least one stimulant prescription fill, by sex, age group, and calendar year — MarketScan commercial databases, United States, 2016–2021

**FIGURE 2:**
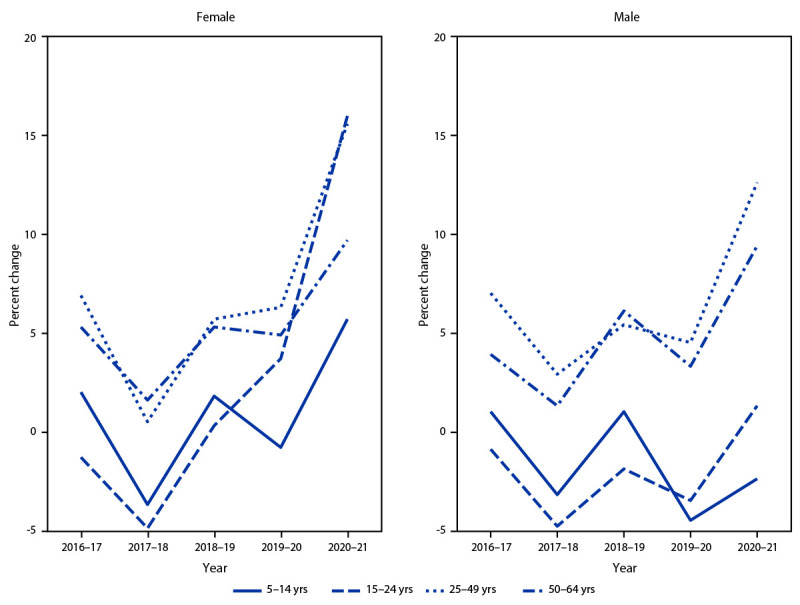
Relative annual percent change in percentage of persons aged 5–64 years with at least one stimulant prescription fill, by sex and age group — MarketScan commercial databases, United States, 2016–2021

During 2016–2020, the pattern among males was similar to that among comparably aged females: the percentage with prescription stimulant fills decreased slightly among those aged ≤24 years (average APC range = –3.8% to –1.7%) and remained stable or increased modestly among those aged ≥25 years (average APC range = 0% to 6.5%). During 2020–2021, the percentage of males with prescription fills decreased among those aged ≤19 years and increased substantially among those aged 25–44 years and 50–54 years (APC range = 11.1% to 14.7%).

Among persons with one or more prescription stimulant fills, the annual mean number of fills ranged from 7.4 to 7.6 (Supplementary Table 1, https://stacks.cdc.gov/view/cdc/125800). Most persons aged 5–19 years (≥75%) and adults aged 20–64 years (53%–77%) with one or more prescription stimulant fills met the case definition for receipt of ADHD care in the preceding or current calendar year; these percentages were relatively stable during the study period (Supplementary Table 2, https://stacks.cdc.gov/view/cdc/125800).

## Discussion

The percentage of persons with employer-sponsored insurance who received prescription stimulants increased during 2016–2021, with notable increases among adolescent and adult females and adult males. The largest single-year increases occurred during 2020–2021, with the annual change exceeding 10% in many age groups. Consistently across the study period, most persons with prescription stimulant fills had health care encounters with ADHD diagnosis codes, and persons with prescription stimulant fills averaged more than seven fills per year, suggesting that most were receiving ongoing care for ADHD.

During this study period, the highest percentages of stimulant prescriptions were among males aged 5–19 years, although these percentages decreased over time. Historically, ADHD has been defined as a childhood disorder more common among boys (*3*), but it is increasingly recognized as a potentially lifelong condition that might be underdiagnosed or undertreated in both girls and adults (*3*,*4*). Appropriate diagnosis and effective treatment can help improve functioning for persons with ADHD (*3*); prescription stimulants have demonstrated effectiveness in reducing ADHD symptoms in children and adults (*3*,*4*).

The prevalence of diagnosed ADHD and associated treatment in adults has increased in recent decades (*1*,*2*,*5*). The current study adds to evidence** that the increasing trend in the percentage of adults receiving prescriptions for stimulants has continued during the COVID-19 pandemic, with a notable upturn during 2020–2021. The pandemic has had negative impacts on mental health (*6*,*7*), which might have led to or exacerbated ADHD symptoms. To adapt to the pandemic environment, policy and health system reimbursement changes were implemented, such as expansion of telehealth and easing of the requirement for having an in-person visit with a clinician before receiving a prescription for stimulants or other Schedule II controlled substances^††^ (*8*). The combination of potential increased need and reduced barriers to access prescription stimulants might have encouraged more adults with ADHD symptoms to seek diagnosis and treatment. Although improved access to ADHD care through telehealth during the pandemic might have benefitted some persons with ADHD symptoms, it might have also introduced the potential for inadequate ADHD evaluations and inappropriate stimulant prescribing. Continued evaluation of public health emergency response policies and their use beyond the immediate emergency, such as expanded use of telehealth for prescribing, could increase understanding of long-term benefits or harms of these policies, including whether these policies increase equitable access to mental health care and the parameters needed to promote best practices (*8*).

The large increase in the percentage of adults receiving prescription stimulants during the COVID-19 pandemic draws attention to the need for clinical practice guidelines for ADHD in adults. Well-established professional guidelines for diagnostic procedures and treatment algorithms exist for children and adolescents with ADHD (*3*); however, no similar diagnostic and treatment guidelines for ADHD among adults are available in the United States (*9*). This gap in guidance for adult ADHD care is a public health concern because of challenges associated with the differential diagnosis of ADHD (*4*,*9*) and general inadequate access to mental health providers (*10*) trained to diagnose and manage ADHD. Clinicians from varying specialties are approached for ADHD care, and report differing levels of training and relative comfort with diagnosing and managing ADHD (*1*,*2*,*9*). Stimulants are one type of treatment that can benefit persons with ADHD, but the potential harms associated with these medications, including adverse effects, interactions with other medications, and risk of diversion, misuse, and overdose (*1*–*4*) necessitate judicious prescribing and patient monitoring. Clinical guidelines similar to those developed for children and adolescents by pediatric medical associations could help clinicians provide best practice care for adult ADHD and support their patients to achieve better outcomes.

The findings in this report are subject to at least seven limitations. First, the data were derived from a large convenience sample of persons with employer-sponsored insurance whose health care use patterns might differ from those of persons with other types of insurance or no insurance. Second, the data do not include the necessary demographic information to examine these trends by race and ethnicity, socioeconomic status, or other characteristics beyond sex and age, in which differences in equity might exist. Third, prescribing policy changes related to the pandemic varied by state (*8*) and might have differential effects, but state-level results are not reported here. Fourth, these results are based on insurance claims, and will not include medications or other ADHD care procured out-of-pocket or obtained through other means. Fifth, the claims data do not include information on the presence of or changes in ADHD symptoms, environmental changes that might have influenced impairment, access to diagnosis and treatment, quality of care, prescribing provider type, or if stimulants were prescribed to treat something other than ADHD; these factors might have varied throughout the study period. In addition, these data do not contain information on whether the encounter during which the prescription was made occurred via telehealth; therefore, the changes in stimulant prescribing patterns described in this study cannot be directly attributed to changes in telehealth availability and related policies. Sixth, because diagnosis codes are not included on prescription drug claims, it cannot be assumed that all prescription stimulants were prescribed to treat ADHD. However, fills for any ADHD medication, including prescription stimulants, were included as part of the case definition for ADHD care. Finally, APC is sensitive to baseline percentage; small absolute fluctuations in groups with lower baseline percentages will result in larger relative percent changes; thus, APC should be interpreted with caution when comparing across groups.

The percentage of persons receiving prescription stimulant fills increased during 2016–2021, including large increases during 2020–2021 and among adolescent and adult females and adult males. These results could guide continued monitoring of and research concerning factors contributing to increases in stimulant prescribing and other changes in care for ADHD symptoms before and during the pandemic, and how they might differ among adults and adolescent females. This study also suggests a growing need for resources to help clinicians accurately diagnose, manage, and treat adults with ADHD. The development and implementation of clinical practice guidelines for adult ADHD could be one component of an approach to facilitating the provision of high-quality care to adults with ADHD.

**Summary**

**What is already known about this topic?**

Prescriptions for stimulants, primarily used to treat attention-deficit/hyperactivity disorder (ADHD), were increasing for adults before the COVID-19 pandemic. Policies enacted during the pandemic expanded access to prescription stimulants via telehealth.

**What is added by this report?**

The percentage of adolescent and adult females and adult males receiving prescription stimulant fills increased during 2016–2021, particularly during 2020–2021.

**What are the implications for public health practice?**

Growing recognition of ADHD in adults and increases in prescription stimulant fills raise questions about current adult ADHD care. Development of clinical recommendations for diagnosing and managing adult ADHD could help guide safe and appropriate stimulant prescribing. Evaluation of policies enacted during the pandemic could identify benefits and harms of those policies.

## Footnotes

*
https://chadd.org/for-professionals/clinical-practice-guidelines/

† Prescription stimulants included in this analysis were amphetamine and mixed amphetamine salts, dexmethylphenidate, dextroamphetamine, lisdexamfetamine, methamphetamine, and methylphenidate.

§ To meet the case definition for receipt of ADHD care, enrollees were required to have two or more health care encounters with an ADHD *International Classification of Diseases, Ninth Revision, Clinical Modification* diagnosis code of 314.X or an *International Classification of Diseases, Tenth Revision, Clinical Modification* diagnosis code of F90 occurring ≥7 days apart, or one or more visits with an ADHD diagnosis code and two or more prescriptions for ADHD medications (any stimulant included in the analysis or atomoxetine, clonidine, or guanfacine) filled ≥14 days apart; these criteria had to be met during the preceding or current calendar year.

¶ 45 C.F.R. part 46, 21 C.F.R. part 56; 42 U.S.C. Sect. 241(d); 5 U.S.C. Sect. 552a; 44 U.S.C. Sect. 3501 et seq.

**
https://www.trillianthealth.com/insights/the-compass/sharp-uptick-in-adderall-prescribing-for-adults-ages-22-44-amid-covid-19-pandemic

††
https://www.kff.org/womens-health-policy/issue-brief/opportunities-and-barriers-for-telemedicine-in-the-u-s-during-the-covid-19-emergency-and-beyond/
